# High-Precision Hysteresis Sensing of the Quartz Crystal Inductance-to-Frequency Converter

**DOI:** 10.3390/s16070995

**Published:** 2016-06-28

**Authors:** Vojko Matko, Miro Milanović

**Affiliations:** 1 Institute for Automation, Faculty of Electrical Engineering and Computer Science, University of Maribor, Smetanova 17, Maribor 2000, Slovenia; 2 Institute for Robotics, Faculty of Electrical Engineering and Computer Science, University of Maribor, Smetanova 17, Maribor 2000, Slovenia; miro.milanovic@um.si

**Keywords:** inductance-to-frequency converter, hysteresis, sensor switching method, inductance, temperature compensation

## Abstract

A new method for the automated measurement of the hysteresis of the temperature-compensated inductance-to-frequency converter with a single quartz crystal is proposed. The new idea behind this method is a converter with two programmable analog switches enabling the automated measurement of the converter hysteresis, as well as the temperature compensation of the quartz crystal and any other circuit element. Also used is the programmable timing control device that allows the selection of different oscillating frequencies. In the proposed programmable method two different inductances connected in series to the quartz crystal are switched in a short time sequence, compensating the crystal’s natural temperature characteristics (in the temperature range between 0 and 50 °C). The procedure allows for the measurement of the converter hysteresis at various values of capacitance connected in parallel with the quartz crystal for the converter sensitivity setting at selected inductance. It, furthermore, enables the measurement of hysteresis at various values of inductance at selected parallel capacitance (sensitivity) connected to the quartz crystal. The article shows that the proposed hysteresis measurement of the converter, which converts the inductance in the range between 95 and 100 μH to a frequency in the range between 1 and 200 kHz, has only 7 × 10^−13^ frequency instability (during the temperature change between 0 and 50 °C) with a maximum 1 × 10^−11^ hysteresis frequency difference.

## 1. Introduction

Nowadays, inductance-to-frequency conversion has become increasingly used in a large variety of applications that are designed for the measurement of a number of physical measurands, such as mechanical displacement (position), pressure, nanopositioning, strain sensing, eccentric motion, biosensors, in medical and electromagnetic material properties measurements, and quartz crystal microbalance [1,2,3]. High-resolution inductance-to-frequency conversion, in particular, is a well-established technique in microscale converters for material properties sensing. It represents a universal transduction mechanism for the measurements in which the inductance changes need to be measured with great precision. The hysteresis of the converter in such measurements plays a very important role.

Recent research studies have focused mainly on the sensor methods that would make precise measurements with the help of an inductance change in the range well below some μH possible. In such measurements both inductive resolution and inductance-to-frequency converter hysteresis play a vital role. The problem of temperature drift was addressed by one of the studies with an analog-digital mixed-measurement method based on the two-dimensional look-up table. Designed for inductive proximity sensors (IPS) widely used in position detection, the method reduced the measurement error caused by temperature drift, but unlike this study it works in the mH inductive range and is not linear [4]. Change in the sensor’s resonant frequency can also be used to detect the pressure wirelessly. In the implantable passive LC pressure sensor proposed by an interesting study, the inductance and capacitance elements of the sensor were designed independently and separated by a thermally-insulating material, which was conducive to reducing the influence of the temperature on the inductance element of the sensor. The linearity and repeatability errors achieved were 95.3%, and 5.5%, respectively, as a function of pressure at 800 °C [5]. Apart from temperature, passive LC sensors can be used to monitor pressure and harmful gases in harsh environments. The paper discusses the advantages and disadvantages of various sensor types and establishes that, for the measurement of multifunction sensors (because of the mutual inductance among multiple parameters), precise readout of multiple parameters requires a complex decoupling method. In addition, current passive LC sensors exhibit some degree of temperature drift [6]. With silicon’s better thermal material properties, silicon micromachined pressure sensors are widely used. The thermal mismatching between the sensing element and packaging may generate stresses on the transducer of a sensing element and create thermal hysteresis and voltage shift during temperature cycling (a very low voltage is involved). In the paper, finite element analyses and experimental tests were conducted to reduce the thermal stress and thermal hysteresis for differential pressure sensors [7].

Other research used a displacement-to-frequency transducer based on the variation of a coil inductance when a magnetic core is partially or completely inserted inside. Studies of the thermal stability of the transducer have been performed with the maximum frequency variation of 24 Hz-equivalent to 21 μm (below the sensor accuracy 33.5 Hz-equivalent to 28 μm). However, no temperature analysis of the influence of the flexible core on the frequency variation was made [8]. The sensing principle of an ink-jet printed eddy current position sensor has been thoroughly studied and validated by a paper that used both simulations and measurements. The results showed that the design of the sensor is very dependent on the limitations of the fabrication, especially the printing of conductors. Additionally, the temperature influence of the oscillator has not been taken into account [9]. Another paper presents a temperature and strain monitoring system for a magnetic actuator based on the magnetostrictive material Terfenol-D (Tb0.3 Dy0.7Fe1.92) with four fiber Bragg sensors. This system allows making the appropriate hysteresis compensation on the strain measurement due to temperature drift. The paper, however, does not analyze the temperature hysteresis influence of the electronic amplifier circuit and fiber Bragg sensor [10]. Small inductance changes measured at the frequency of 4.999 MHz can be detected with high sensitivity in the temperature range between 10 °C to 40 °C. The main advantage of this method lies in a considerable reduction of the temperature influence of the AT-cut crystal frequency change in the temperature range between 10 °C and 40 °C through a switching method. The method, however, has not researched the hysteresis influence brought about by the switching method [11]. The above methods looked for ways either of how to detect temperature influence or how to compensate it, but they have not made any significant analysis with regard to the sensors’ (electronic circuits) hysteresis influence on the measurement error.

The novelty in this work is the automated measurement of the quartz crystal inductance-to-frequency converter characteristics and hysteresis through the switching between the measuring inductance and the reference one using an analog multiplexer (MUX) switch. To set the capacitance in the first place, an analog single-pole single-throw (SPST) switch for the switching of additional parallel capacitances to the quartz crystal is used. In addition, a programmable switching control device for the converter hysteresis measurement was added. The use of all of these elements improves the quality of converter characteristics and hysteresis measurement, the frequency sensitivity of the converter, compensates the quartz crystal non-linear self-temperature frequency characteristic, as well as any parasitic capacitances of the oscillator circuit, and enables a very stable converter functioning in an extended temperature range. A switching mode oscillator, furthermore, strongly reduces the influence of the supply voltage on the output signal oscillating frequency, and foresees the functioning of the sensitive inductance element.

Additional comparisons to some other methods [12,13,14] for the conversion of inductance to frequency and hysteresis detection reveal that the newly-proposed method also proves to have high dynamic stability during the temperature changes in the extended operating range when the temperature varies between 0 and 50 °C. With regard to the temperature, the method makes possible a stable functioning of small inductance change measurement and conversion to a frequency signal (without any additional lock-in amplifier, host system, or temperature sensor).

## 2. Switching Mode Inductance-to-Frequency Converter

### 2.1. Switching Converter Principle

A quartz crystal inductance-to-frequency converter uses the principle of switching between two inductances connected in series, changing in this way the quartz crystal series resonance frequency. The purpose of this switching is the temperature compensation of the serial resonance frequency, which is temperature-dependent due to the quartz temperature dependency. It additionally compensates any other influence, such as parasitic capacitances and inductances, aging of the elements, etc., with the help of an additional reference inductance [11].

**Figure 1:**
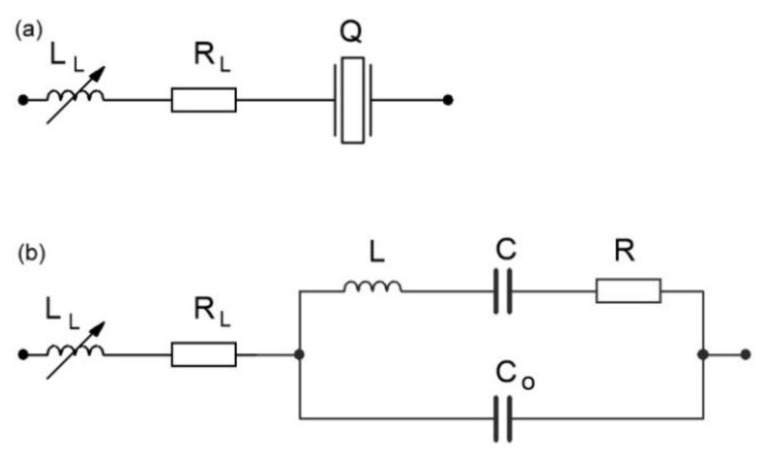
(**a**) The real inductance *L*_L_ + *R*_L_ in series with the quartz crystal and (**b**) the real inductance *L*_L_ + *R*_L_ with the quartz crystal equivalent circuit.

The operation of a quartz crystal (frequently explained using the familiar “Equivalent Circuit”) is illustrated in Figure 1a,b representing an electrical depiction of the quartz crystal unit [15,16,17]. By connecting the inductance *L*_L_ with its own resistance *R*_L_, the quartz crystal series resonance frequency can be changed from 0 to 200 kHz.

The quartz series resonance frequency without the inductance *L*_L_ (which has its own resistance *R*_L_) connected in series is determined by Equation (1):

(1) $$f_{0}=\frac{1}{2\pi \cdot \sqrt{L\cdot C}}$$

For the connection of the quartz crystal with inductance *L*_L_ in series, an equation with equivalent impedance can be written. If we define the frequency ratio Ω = ω/ω_o_, which depends on ω_o_ = 1/$\sqrt{L\cdot C}$, and take into account ω_o_ *L* = 1/ω_o_ *C*, then the impedance change in the vicinity of the resonance frequency is described by Equation (2) in the range of change Ω = 0.998–1.038 [15,16,17]:

(2) $$\bar{Z}\left(\Omega \right)=R\frac{1+j\frac{\omega_{0}L}{R}\left(\Omega -\frac{1}{\Omega }\right)}{1+\frac{C_{0}}{C}\left(1-\Omega^{2}\right)+j\frac{C_{0}}{C}\cdot \frac{R}{\omega_{0}L}\cdot \Omega }+j\frac{\Omega \cdot L_{L}}{\sqrt{L\cdot C}}+R_{L}$$

**Figure 2:**
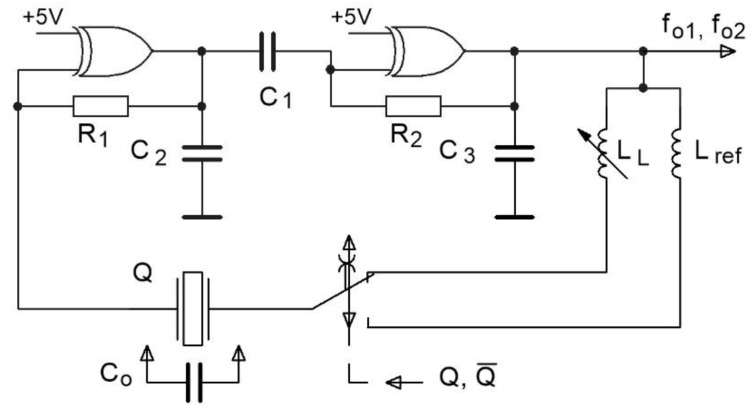
Switching of the oscillator inductances in the inductance-to-frequency converter.

Figure 2 depicts the oscillator circuit switching between the reference inductance *L*_ref_ in series with the quartz crystal and the measuring inductance *L*_L_ in series with the quartz crystal. The switching between these two inductances produces two new resonance frequencies *f*_01_ and *f*_02_ at the output. Switching signals Q and $\;\bar{Q}$ are digital signals 1 or 0.

The pulling range between the switching of inductances *L*_L_ and *L*_ref_ with the signals Q and $\bar{Q}$ (Figure 2) can be written with Equation (3) [15,16,17]:

(3) $$D_{L_{L},\;L_{ref}}=\frac{f_{01L_{L}}-f_{02Lref}}{f_{02Lref}}$$

The change of resonance frequency *f*_01_ (Figure 2) produced by the influence of inductance *L*_L_ is expressed with Equation (4) [15,16,17]:

(4) $$f_{01}=\frac{1+\frac{C}{2\left(C_{0}-\frac{1}{{\omega_{0}}^{2}\cdot L_{L}}\right)}}{2\pi \cdot \sqrt{L\cdot C}}\;$$

The change of resonance frequency *f*_02_ (Figure 2) produced by the influence of inductance *L*_ref_ is described in Equation (5) [15,16,17]:

(5) $$f_{02}=\frac{1+\frac{C}{2\left(C_{0}-\frac{1}{{\omega_{0}}^{2}\cdot L_{ref}}\right)}}{2\pi \cdot \sqrt{L\cdot C}}\;$$

### 2.2. Converter Hysteresis Measurement Principle

**Figure 3:**
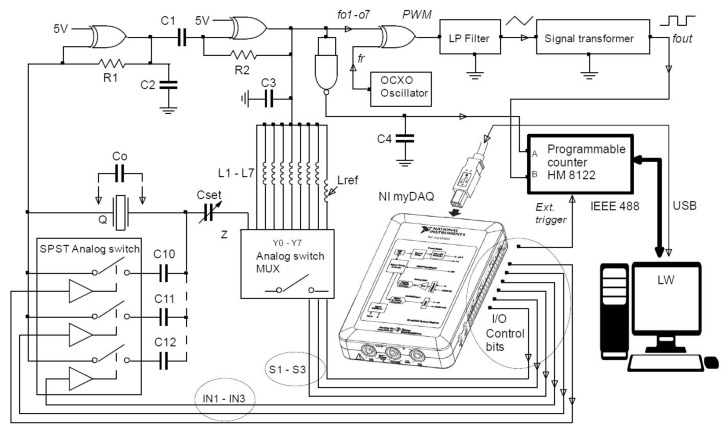
Schematic representation of the inductance-to-frequency converter hysteresis measurement.

The new idea of the converter hysteresis measurement method described in this article is the use of a specific switching mode oscillator with additionally-connected inductances in series with the quartz crystal alternating between *L*_1_–*L*_7_ and *L*_ref_ (Figure 3). Additional shunt capacitances *C*_10_–*C*_12_ are connected together with the SPST analog switch in parallel to the quartz crystal for the experimental stepwise increase (sensitivity settings) of the capacitance *C*_0_. Conversion inductances *L*_1_–*L*_7_ and reference inductance *L*_ref_ are connected to the quartz crystal alternately (switching mode) in series. They enable a significant reduction of the temperature influence (both are on the same temperature) on the oscillator frequency change because of the switching mode oscillator. Capacitance *C*_set_ is used here for accurate setting of the initial frequency of the oscillator. To switch the frequencies from *f*_01_ to *f*_07_ the NI myDAQ (National Instruments, Austin, TX, USA) programmable device and the LabVIEW (LW) software (National Instruments, Austin, TX, USA) are used. The switching is performed through the switching (switching table of the eight-channel analog MUX switch) of digital signals (wires S_1_–S_3_). With the help of the reference frequency *f*_r_ (from an oven-controlled crystal oscillator (OCXO18T5S)), pulse width module (PWM), and low-pass filter (LP) frequencies *f*_01–07_ (≅4 MHz) are converted to the range between 1 and 200 kHz, which is suitable for further signal processing. The signal corresponding to the frequency difference between the oscillator frequencies *f*_01–07_ and reference frequency *f*_r_ enters the LP filter (which is a pulse width modulated signal) [18,19,20]. At the passive LP filter (with the response time of 3 μs) output, the triangular signal (with the initial setting frequency of 5 kHz depending on *C*_set_, *L*_ref_, and *f*_r_ = 4 MHz) is produced (frequency difference between *f*_0Lref_ and *f*_r_). This signal is then converted to a rectangular signal by a signal transforming circuit representing the output signal *f*_out_ of the inductance-to-frequency converter. The output frequency *f*_out_ thus represents the temperature and any other influence-compensated signal which is synchronously measured with regard to the switching frequency. The latter can change in the range *f*_Switch_ = 1–20 Hz [10]. Capacitances *C*_2_ and *C*_3_ serve to suppress the spurious responses to avoid crystal oscillation at higher frequencies, and *C*_4_ reduces the high frequency noise of the oscillator in the channel A of the HM 8122 programmable counter [21]. Channel A of the programmable counter measures the frequency *f*_01-07_ in relation to the digital signals (wires S_1_–S_3_), while channel B measures the frequency difference *f*_out_ = *f*_01-07_ − *f*_r_ or *f*_0Lref_ − *f*_r_ depending on the switching digital state (wires S_1_–S_3_).

For every switching between one of the inductances *L*_1–7_ and *L*_ref_ (by changing digital signals sent by wires S_1_–S_3_), a positive impulse from NI myDAQ triggers the frequency measurement on the counter (external trigger). In this way, the frequency counter measures frequency synchronously with the LW programmable digital signal changes (wires S_1_–S_3_). Moreover, the LW software driver also makes it possible to statistically evaluate the number of performed consecutive measurements.

The quartz stray capacitance *C*_0_ comprises pin-to-pin parallel capacitance at the crystal pins, plus any parasitic capacitances. The typical value of the quartz stray capacitance is between 1.5 pF and 5 pF. The connection of an additional stray capacitance connected in parallel (by SPST analog switch controlled by digital signals sent by wires IN_1_–IN_3_) to the quartz crystal for capacitive load settings, and of inductance *L*_1–7_ in series with the quartz crystal expands the possibility of the measurement of the inductance-to-frequency converter hysteresis where the stable oscillation and high sensitivity are one of this method’s major advantages [22,23,24].

### 2.3. Temperature Compensation

Temperature compensation of the inductance-to-frequency converter is achieved via the oscillator’s switching method, which reduces the influence of short-term temperature instability and long-term instability (aging), representing the oscillator frequency variation as a function of time. The short-term stability of a quartz crystal depends on the actual oscillator design and is totally controlled by the quartz crystal at a low drive level (<10 μW) [25]. Long-term stability changes with years and is naturally greater during the first part of the crystal unit life. The aging rates of the best cold weld crystals are less than ±1 ppm/year (0–50 °C) [22].

The principle of temperature compensation is explained below through the example of two switchings between the inductances *L*_L_ and *L*_ref_ (as illustrated on Figure 2), where inductance *L*_L_ represents the inductance *L*_1_ in Figure 3 [11]. When inductances *L*_1_ and *L*_ref_ are the same, *f*_01_ and *f*_02_ remain almost the same depending on the quartz crystal resonant frequency *f*_0_, quartz crystal temperature characteristics *Δf*_0_ (*T*), its aging *Δf*_0_ (*t*), and the *L*_1_ and *L*_ref_ inequality, as well as the *Δf*_0_ (*ΔC*_0p_) change. However, when inductances *L*_1_ and *L*_ref_ are different, the frequencies *f*_01_ and *f*_02_ are different and depend on the quartz crystal series resonant frequency *f*_0_, quartz crystal temperature characteristics *Δf*_0_ (*T*), its aging *Δf*_0_ (*t*), inductances *Δf*_0_ (*L*_2_) and *Δf*_0_ (*L*_ref_), as well as the *Δf*_0_ (*ΔC*_0p_) change. In the case of the difference of the two frequencies *f*_01_ and *f*_02_, *Δf*_0_ (*T*), *Δf*_0_ (*t*), and *Δf*_0_ (*ΔC*_0p_) are strongly reduced because only one temperature quartz characteristic is involved [11].

*f*_0_—quartz crystal series resonant frequency

*f*_r_—reference frequency

Δ*f*_c_—counter error

*L* and *C—*mechanical behavior of the crystal element

*L*_n_—conversion inductance (*n* = 1–7)

*L*_ref_—reference inductance

*C*_0_—quartz crystal parallel capacitance

*C*_0p_—all actual parallel parasitic capacitances connected to the quartz crystal

*T—*temperature

*t—*time

The output frequency *f*_out_ (Figure 3) depends on the selection of digital signals sent by wires S_1_–S_3_ (frequency *f*_01-07_) and reference frequency *f*_r_ and can be expanded to (in case *Z*_1_ = *R*_1_ + j*ωL*_1_ to *Z*_7_ = *R*_7_ + j*ωL*_7_ and *Z*_ref_ = *R*_ref_ + j*ωL*_ref_, whereby when dealing with small inductance values, resistances *R*_1-7_ and *R*_ref_ can be ignored):

(6) $$\;f_{out1}=f_{01}-f_{r}=f_{0}+\Delta f_{0}\left(T_{1}\right)+\Delta f_{0}\left(t_{1}\right)+\Delta f_{0}\left(C_{0p}\right)+\Delta f_{0}\left(L_{n}\right)-(f_{r}\left(T_{1}\right)\;+\Delta f_{r}\left(T_{1}\right))+\Delta f_{c1}\left(t_{1}\right)$$

(7) $$\;f_{out2}=f_{02}-f_{r}=f_{0}+\Delta f_{0}\left(T_{2}\right)+\Delta f_{0}\left(t_{2}\right)+\Delta f_{0}\left(C_{0p}\right)+\Delta f_{0}\left(L_{ref}\right)-(f_{r}\left(T_{2}\right)\;+\Delta f_{r}\left(T_{2}\right))+\Delta f_{c2}\left(t_{2}\right)$$

where *Δf*_r_ (T) in Equations (6) and (7) represents the temperature instability of the reference oscillator signal and *Δf*_c_ (*t*) a counter error [16,17]. The joining of *f*_0_ and *Δf*_0_ (*L*_n_) gives Equation (8). Which represents *f*_01_:

(8) $$f_{01}=\frac{1+\frac{C}{2\left(C_{0p}-\frac{1}{{\omega_{0}}^{2}\cdot L_{n}}\right)}}{2\pi \cdot \sqrt{L\cdot C}}\;+\Delta f_{0}\left(T_{1}\right)+\Delta f_{0}\left(t_{1}\right)$$

(9) $$\omega_{0}=2\pi f_{0}$$

The joining of *f*_0_ and *Δf*_0_ (*L*_ref_) gives Equation (10) which represents *f*_02_:

(10) $$f_{02}=\frac{1+\frac{C}{2\left(C_{0p}-\frac{1}{{\omega_{0}}^{2}\cdot L_{ref}}\right)}}{2\pi \cdot \sqrt{L\cdot C}}+\Delta f_{0}\left(T_{2}\right)+\Delta f_{0}\left(t_{2}\right)$$

At every switch between two inductances, the frequency *f*_out_ is measured synchronously by the HM 8122 programmable counter (Figure 3) and its value is transferred to the LW software calculating the difference between the two frequencies. This gives the frequency difference in Equations (11) and (12), representing the temperature-compensated value of the output frequency *f*_out_ , depending almost uniquely on the difference between the *ΔL*_n_ and *ΔL*_ref_ change. This means that *Δf*_out_ (*L*_n_) is virtually independent of the quartz crystal temperature characteristics *Δf*_0_ (*T*). The quartz aging *Δf*_0_ (*t*) is practically compensated and can be ignored as the measurements are short and consecutive (a few milliseconds). Due to the switching of the frequencies *f*_01_ and *f*_02_, the output frequency *f*_out_ also highly reduces the auxiliary reference frequency *f*_r_ temperature instability *Δf*_r_ (*T*):

(11) $$\Delta f_{out}\left(L_{n}\right)=\left[f_{01}-(f_{r}\left(T_{1}\right)+\Delta f_{r}\left(T_{1}\right)+\Delta f_{c1}\left(t_{1}\right))\right]\;-\left[f_{02}-(f_{r}\left(T_{2}\right)+\Delta f_{r}\left(T_{2}\right)+\Delta f_{c2}\left(t_{2}\right))\right]$$

The reference frequency *f*_r_ is OCXO18T5S oven-controlled crystal oscillator (4 MHz) with the frequency stability of ±0.01 ppm in the temperature range 0° ± 60 °C following the warm-up time of 1 min [25,26,27]. The *f*_r_ (*T*) is compensated in Equation (11). Equation (12) is formed taking into account that the counter error *Δf*_c_ (*t*) is different at every switching for the time *t*_1_ and *t*_2_:

(12) $$\Delta f_{out}\left(L_{n}\right)=\left[\frac{\frac{C}{2\left(C_{0p}\;-\frac{1}{{\omega_{0}}^{2}\cdot L_{n}}\right)}}{\begin{array}{c} 2\pi \cdot \sqrt{L\cdot C} \end{array}}\right]-\Delta f_{0}\left(T_{1}\right)-\Delta f_{r}\left(T_{1}\right)+\Delta f_{c1}\left(t_{1}\right)\;-\left[\frac{\frac{C}{2\left(C_{0p}\;-\frac{1}{{\omega_{0}}^{2}\cdot L_{ref}}\right)}}{\begin{array}{c} 2\pi \cdot \sqrt{L\cdot C} \end{array}}\right]+\Delta f_{0}\left(T_{2}\right)+\Delta f_{r}\left(T_{2}\right)-\Delta f_{c2}\left(t_{2}\right)$$

The proposed method (Figure 3) allows the AT-cut crystal temperature characteristics compensation (under 0.00001 Hz) in the range between 0 and 50 °C for the crystal cut angle 0′ through the switching circuit and significantly reduces its influence to a minimum.

### 2.4. The Shortcoming of the Temperature Compensation

The temperature compensation should take into account that due to the converter switching mode, *L*_n_ and *L*_ref_ are “alternatively” connected in series to the crystal in the oscillator in various sequences. The frequencies *f*_01_ and *f*_02_ given by Equations (8) and (10) have different times *t*_1_ and *t*_2_ (one after the other) depending on the digital control signals (wires S_1_–S_3_). This means that the subtraction in Equation (12) is not performed exactly point-to-point in time. It should be mentioned that the approach has some limitations in terms of the switching times and the time-speed of the events, i.e., temperature changes, whose effects can be cancelled. If these changes are sufficiently steep, temperature-related terms (*Δf*_0_ (*T*_1_) and *Δf*_0_ (*T*_2_) in Equations (8) and (10) are not equal, so they are not fully counterbalanced in Equation (12)) may not be cancelled in Equation (8). As a result, the lineal first-order approximations of Equations (6) and (7) are no longer valid, which means that the influence of other terms is also non-negligible.

Frequency changes of the counter error Δ*f*_c1_ (*t*) and Δ*f*_c2_ (*t*) in Equations (6) and (7) are undefined, and presented here as a part of these equations. They, in fact, include both the counter measurement error and oscillator noise because it is very difficult to differentiate between the two. The frequency measurement errors can differ in every single measurement. Different oscillator noises (phase modulated (PM), jitter, and thermal Johnson) are all included in Δ*f*_c_ (*t*) [21]. The switching mode method, first and foremost, compensates (considerably reduces) quartz crystal temperature influence. This influence is significantly greater than those of the noise and counter accuracy.

The frequency measurement time depends on the gate time of the HM 8122 programmable counter and that of the LW software, as well as on the speed of the instrumentation IEEE 488 interface bus. To generate digital control signals (S_1_–S_3_) and perform synchronous measurements, an additional electronic circuit NI myDAQ (Figure 3) was added. The switching signal switching between *f*_01_ and *f*_02_ is simultaneously used as an external signal triggering the HM 8122 programmable counter (frequency measurement (channel A and channel B). The counter synchronously measures the sequence frequency *f*_01_ (channel A) (the time of one measurement is determined by the counter gate time, which cannot be less than 1 ms) and frequency *f*_out_ (channel B). Similarly, the frequencies *f*_02_ and *f*_out_ are sequentially measured on the counter channel A and B in the next switching sequence, and LW software calculates the frequency difference between Equations (8) and (10). For every two frequencies measured by the counter, LW software calculates the frequency difference (Equation (12)).

The maximum variation temperature/time (*ΔT/Δt*) limit for which the compensation is still achieved is determined by the response times of the converter, LW software, and HM 8122 counter measurement time. Converter response *f*_0_ versus inductance variation is determined by the eight-channel analog MUX switching time (the values for the ON and OFF mode are 59 ns and 60 ns, respectively), and the low-pass filter time constant, which is 3 μs (passive LP filter first-order (τ = *R*_LP_ · *C*_LP_)). If we take into account the response time of the two switches for one temperature-compensated inductance measurement, the converter response time is ≥10 μs. Due to LW software communication with the HM 8122 programmable counter and the time needed for the measurement (the time for one frequency measurement in the channel A or B is 1 ms) of the two frequencies by the counter, the minimum response time is not less than 2.1 ms. The maximum variation temperature/time *(ΔT/Δt*) limit for which the compensation is still achieved is determined by the dynamic frequency measurement error value during the time of both *f*_01_ and *f*_02_ signal period, i.e., within 2.1 ms (two consecutive measurements).

### 2.5. Experimental Setup

**Figure 4:**
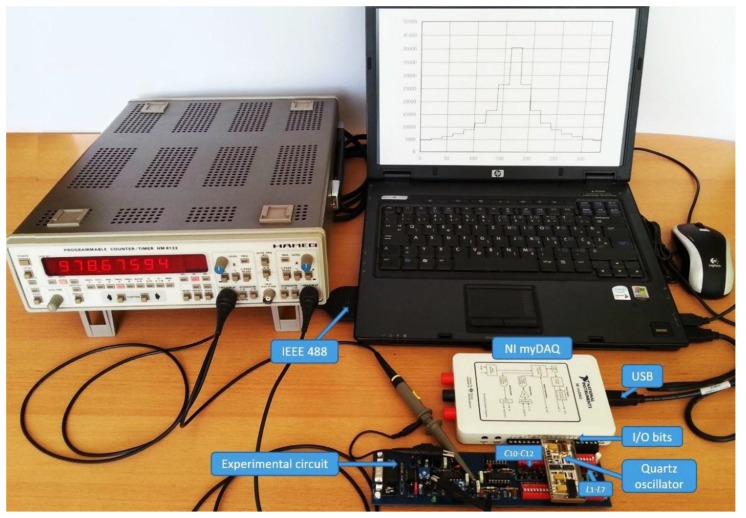
Experimental setup with experimental switching circuit.

In this experiment, a prototype of a switching inductance-to-frequency converter circuit was constructed guaranteeing physically-stable conditions of the converter elements (Figure 4). A control switching logic was applied to the experimental oscillator circuit (eight-channel analog switch MUX) for the switching of the inductance *L*_1–7_, *L*_ref_ and capacitance (SPST analog switch) *C*_10_–*C*_12_ (Figure 3). The circuit also includes an OCXO oscillator (Figure 4), producing a frequency-stable reference rectangular signal, and pulse-width modulator (XOR logic gate) producing at the output a pulse-width modulated signal. A low-pass filter filters the frequency difference *f*_01_–*f*_r_ in the first logic state of the switching (S_1_–S_3_) and the frequency difference *f*_02_ − *f*_r_ in the second logic state of the switching (S_1_–S_3_). The signal transformer transforms the voltage triangular signal to the voltage rectangular signal. The quartz oscillator is produced with SMD technology on Al_2_O_3_ because of good temperature stability and is placed into a metal housing. This design was used to achieve as stable parasitic capacitances and inductances in the circuits as possible. The quartz crystal Q (Figure 3) used in the experiment was AT-cut crystal with a frequency change of −2.5 ppm at *T* = 0 °C and 1.5 ppm at *T* = 50 °C depending on the reference temperature point *T*_ref_ = 25 °C. The data of the electrical quartz crystal (*f*_0_ = 4 MHz) equivalent circuit elements are *R* = 10 Ohm, *C* = 25 fF, *L* = 64 mH, *C*_o_ = 4 pF, and quality *Q* = 80 k. The values in the quartz crystal equivalent circuit were measured by an HP4194A impedance/gain-phase analyzer. Experimental circuits is connected to the NI myDAQ device with digital outputs for the control of the eight-channel analog switch MUX and a SPST analog switch. The NI myDAQ device is further connected to the computer via USB port. This enables automated changing of inductances *L*_1–7_ at the input of the inductance-to-frequency converter and switching between *L*_1–7_ and *L*_ref_ and the setting of the converter sensitivity with capacitances *C*_10_–*C*_12_ (Figure 3). This mode of operation ensures stable parasitic capacitances and inductances, as well as repeatability of the experimental results [28,29].

The HM 8122 programmable counter is connected to the computer via the IEEE 488 interface bus. The measurement probes are connected directly to output pins *f*_out_ and *f*_01–07_ of the experimental circuit. Moreover, the LW software performs the switching of the oscillator frequency from *f*_01_ to *f*_07_ via NI myDAQ device, reads the frequency data for each *f*_0n_ from the counter via the IEEE 488 interface bus, and processes the data.

## 3. Experimental Results

To obtain good experimental results, a stable oscillator circuit in the inductance-to-frequency converter is of crucial importance. Therefore, the factors affecting frequency stability of the converter, such as wide operating temperature range, the use of various types of crystals, and drive level should be considered. Stability of the electronic circuit depends upon the quartz crystal temperature stability, the circuit type and quality of the elements. It is noteworthy to mention that an oscillator with a good start-up, i.e., with a reliable crystal oscillation during the start and later on, is a must.

### 3.1. Inductance-Frequency Characteristics of the Converter

**Figure 5:**
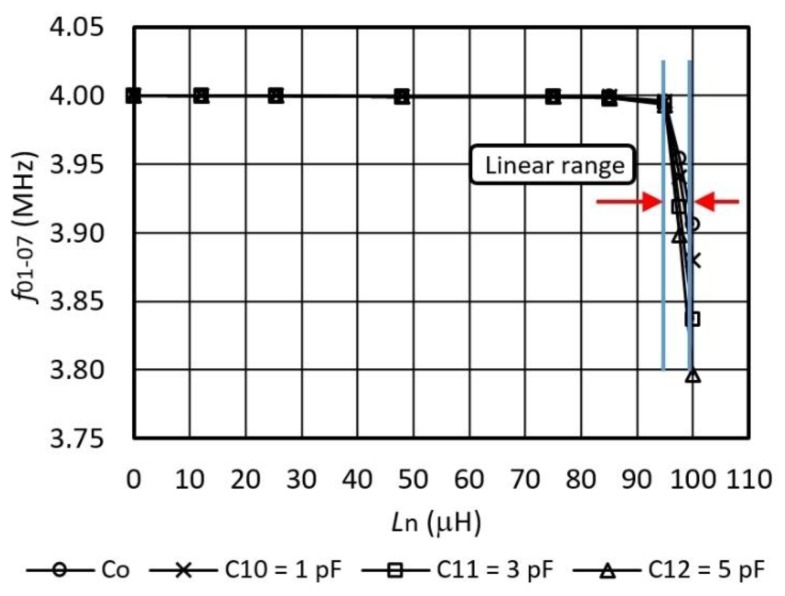
Inductance-frequency characteristics *f*_01-07_ for inductance settings in steps of 0 μH, 12 μH, 25.4 μH, 48 μH, 75 μH, 85 μH, 95 μH, 97.5 μH, and 100 μH (for different values of capacitance *C*_10_–*C*_12_ in parallel to the crystal at *T* = 25 °C).

Figure 5 shows oscillator’s frequency characteristics *f*_01–07_ with regard to the change of the inductance *L*_n_ = *L*_1_ to *L*_7_ and a comparison of the characteristics for various *C*_10_–*C*_12_ (for the selection of various sensitivities) connected in parallel to the quartz crystal Q (Figure 3). *L*_n_ values are set by Ni myDAQ and are in steps of 0 μH, 12 μH, 25.4 μH, 48 μH, 75 μH, 85 μH, 95 μH, 97.5 μH, and 100 μH. Within the switcher range (*L*_n_ = 95–100 μH) the inductance-frequency characteristics is almost linear. In the range *L*_n_ = 95–100 μH, the capacitance *C*_10_ = 1 pF records the sensitivity of 23.12 kHz/μH, *C*_11_ = 3 pF records the sensitivity of 31.58 kHz/μH, and when *C*_12_ = 5 pF, the sensitivity is 39.26 kHz/μH (Figure 5). When only the quartz crystal’s natural *C*_o_ capacitance is present in the circuit, the sensitivity is 17.95 kHz/μH. However, for this particular experiment, the capacitors *C*_10–12_ and inductances *L*_n_ with tolerance of 0.1% were specially selected by the measurement with an HP 4194A impedance/gain phase analyzer.

**Figure 6:**
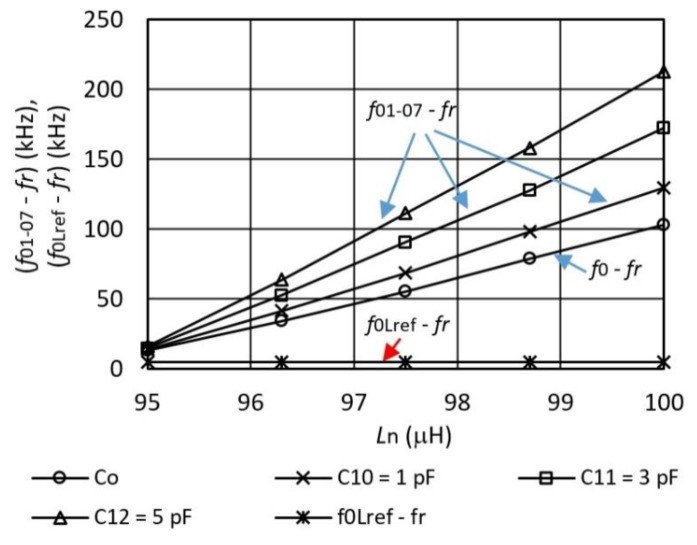
Characteristics *f*0 − *f*_r_, *f*01-07 − *f*_r_ (depending on *L*_n_) and *f*_0Lref_ − *f*_r_ = 5 kHz acquired with the alternate multiplexer switching at different capacitances *C*_10-12_ = 1, 3, and 5 pF at *T* = 25 °C).

Figure 6 shows in greater detail inductance-frequency characteristics in the range of inductance-frequency switcher operation when the inductance is changed in the range *L*_n_ = 95–100 μH. By using a switching multiplexer (and switching signals sent by wires S_1_–S_3_) (Figure 3), where various inductances *L*_n_ are switched, we get characteristics *f*_01–07_ − *f*_r_ and *f*_0Lref_ − *f*_r_. Depicted are three different characteristics for three different capacitance values *C*_10_–*C*_12_, which are switched with the digital signals sent by wires IN_1_–IN_3_ (in the range 1, 3, and 5 pF) and characteristics *C*_0_ (when the crystal does not have any additionally connected capacitance in parallel). Frequency difference *f*_0Lref_ − *f*_r_ = 5 kHz is determined by the reference inductance *L*_ref_ (Figure 3) and the OCXO reference oscillator, and serves for the transformation of the signal in the lower frequency range and for the temperature compensation. Frequency signal *f*_out_ represents the time duration of one switching *f*_01-07_ − *f*_r_ and in the next one *f*_0Lref_ − *f*_r_. The difference of frequencies (*f*_01-07_ − *f*_r_) − (*f*_0Lref_ − *f*_r_) in the sequence of two switchings is then calculated by the computer and represents the final switcher’s inductance-frequency characteristics. Thus, the most sensitive characteristics are obtained at *C*_12_ = 5 pF. The greatest frequency difference of (*f*_01-07_ − *f*_r_) − (*f*_0Lref_ − *f*_r_) at the sensitivity of 39.26 kHz/μH is ≅200 kHz.

**Figure 7:**
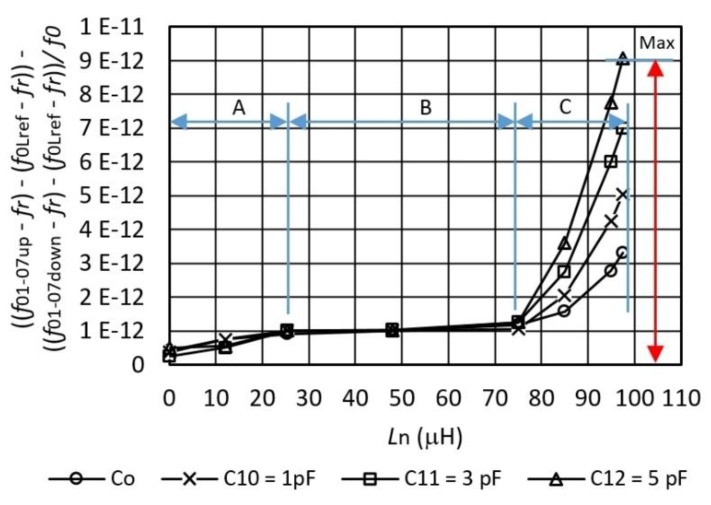
Hysteresis measurement error of the inductance-to-frequency converter depending on the *L*_n_ = 0–99 μH and capacitor value from *C*_10-12_ = 1–5 pF, and switching frequency *f*_switch_ = 1 Hz between *L*_n_ and *L*_ref_.

Figure 7 shows the measurement of the hysteresis error if the inductance *L*_n_ is set in steps between 0 μH and 100 μH (frequencies *f*_01-07up_) and back from 100 μH to 0 μH (frequencies *f*_07–01down_). The influences of the temperature, oscillator element aging, and the influences of parasitic capacitances are reduced to a minimum because in the frequency difference ((*f*_01-07up_ − *f*_r_) − (*f*_0Lref_ − *f*_r_)) − ((*f*_07-01down_ − *f*_r_) − (*f*_0Lref_ − *f*_r_)) remains only (*f*_01-07up_ − *f*_07-01down_), which represents the hysteresis measurement for three capacitances (*C*_10_, *C*_11_ and *C*_12_). Also shown are the hysteresis characteristics for *C*_0_ only, without any additionally-connected capacitances in parallel to the quartz crystal. The hysteresis influence on Figure 7 is visible because, by increasing the value of inductance *L*_n_, the hysteresis (frequency difference (*f*_01-07up_ − *f*_07-01down_)) difference increases. There are three regions A, B, and C shown in Figure 7. In the (A) region, the hysteresis frequency difference for all three capacitance values is *C*_10-12_ ≤ 1·10^−12^, in the (B) region of inductance *L*_n_ = 25–75 μH, the frequency difference is ≅1×10^−12^ and in the (C) region (*L*_n_ = 75–99 μH ) there is a maximum value (Max) of frequency difference at *L*_n_ = 99 μH and it is 9 × 10^−12^ at the value of capacitance *C*_12_ = 5 pF (at maximum sensitivity of the inductance-to-frequency switcher).

### 3.2. Temperature/Frequency Stability of the Switching Converter

**Figure 8:**
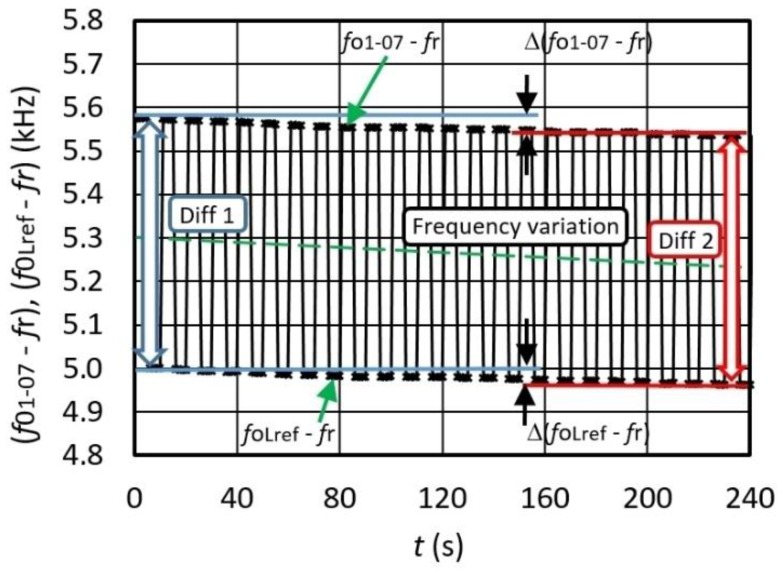
Extended temperature dynamic (switching mode) frequency instability for *f*_01–07_ − *f*_r_ and *f*_0Lref_ − *f*_r_ (sensitivity is 31.58 kHz) (temperature change from 0 to 50 °C).

If the converter is influenced by a temperature changing from 0 to 50 °C, the switching mode extended dynamic frequencies change *f*_01-07_ − *f*_r_ and *f*_0Lref_ − *f*_r_ is approximately the same, and it decreases as shown in Figure 8. For every state *f*_01-07_ − *f*_r_ and *f*_0Lref_ − *f*_r_ consecutive measurements were made. The temperature control was performed by Weiss SB1 160 programmable climate chamber (Weiss Umwelttechnik GmbH, Stuttgart, Germany). The dotted trend line points to the change of direction of both frequency differences (decreases). These results demonstrate that the temperature influence on the frequency differences *f*_01-07_ − *f*_r_ and frequency *f*_0Lref_ − *f*_r_ (in the time span between 0 and 240 s) changes the frequency difference in the same size class (Diff 1, Diff 2). Given that the frequency change is almost the same at 0 °C and 50 °C (Figure 8), the temperature influence is reduced to minimum. The temperature in the immediate vicinity of the crystal was measured by the NI USB-TC01 thermocouple measurement device. The frequency shift shown in Figure 8 between *f*_01-7_ − *f*_r_ and *f*_0Lref_ − *f*_r_ depends on the difference between *L*_n_ and *L*_ref_ (*L*_n_ = 95.01 μH and *L*_ref_ = 95 μH).

**Figure 9:**
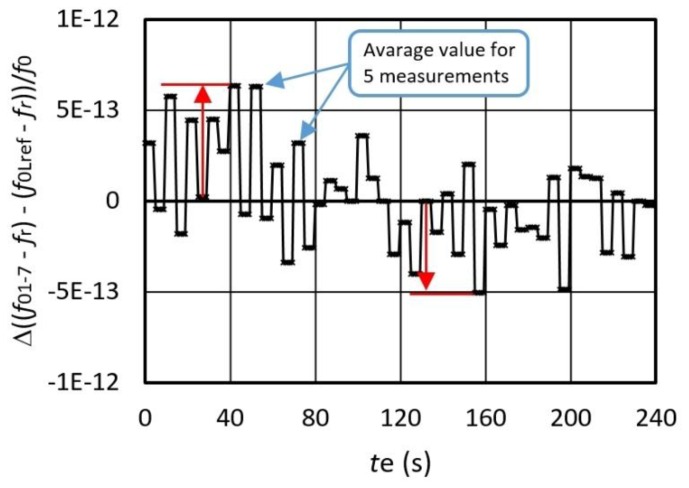
Output frequency variation of Δ*f*_out_ = Δ((*f*_01-07_ − *f*_r_) − (*f*_0Lref_ − *f*_r_))/*f*_0_ during the change of temperature from 0 to 50 °C (*L*_n_ = 95.01μH, *L*_ref_ = 95 μH).

Figure 9 illustrates the frequency variation for Δ*f*_out_ = Δ ((*f*_01-07_ − *f*_r_) − (*f*_0Lref_ − *f*_r_))/*f*_0_ at the frequency difference ≅558 Hz (*f*_0Lref_ − *f*_r_ = 5 kHz (Figure 8)) determined by the fixed values *L*_n_ and *L*_ref_ (*L*_n_ = 95.01 μH, *L*_ref_ = 95 μH) in the time span *t* = 0–240 s during the temperature change 0–50 °C. Deduction of frequencies (between *f*_01-7_ − *f*_r_ and *f*_0Lref_ − *f*_r_) in relation to the synchronization signal is performed by LW software in sequence at different times *t*_e_. The differences between *f*_01-7_ − *f*_r_ and *f*_0Lref_ − *f*_r_ show a high-frequency dynamic stability in the range ±7 × 10^−13^ (as pointed by arrows on Figure 9).

## 4. Discussion

This switching method makes possible a high-precision measurement of accurate characteristics and hysteresis of the inductance-to-frequency converter through experimental automated approach.

Due to almost instant comparison (that lasts only a few milliseconds) of the measured inductance with the reference one, the environment temperature remains virtually unchanged. In case of two consecutive inductance measurements and the conversion to frequency (at dynamic environment temperature changes), the temperature influence is also reduced to a minimum, as shown by the results. The frequency difference of two consecutive switchings namely lasts only a few milliseconds (Figure 8 and Figure 9). An automated approach of changing inductance with the help of an analog MUX switch (Figure 3) ensures that during the switchings there are no additional changing influences of parasitic inductances and capacitances. The SPST analog switch plays a similar role, changing the load capacitance and, as a result, the sensitivity of the inductance-to-frequency converter.

If the output converter frequency sensitivity of 39.26 kHz is in the range *L*_n_ = 95–100 μH at *C*_12_ = 5 pF (Figure 6), the supply voltage stability is 5 V ± 0.01 V, the HM8122 counter accuracy of ±5 × 10^−9^ (through the working temperature range from 10 to 40 °C, and the frequency reference *f*_r_ stability is 0.005 ppm, then the frequency stability at the output is *f*_out_ = ±7 × 10^−13^, which gives the converter a resolution of ±2 pH in the temperature range between 0 and 50 °C. Thus, the output frequency stability proves the applicability of the automated hysteresis measurement method of the inductance-to-frequency converter in the pH range.

## 5. Conclusions

The proposed measurement method enables temperature compensation and stable hysteresis measurement of an inductance-to-frequency converter. The greatest advantage of the proposed method is that the programmable timing control device allows for the selection of different oscillating frequencies. The method simultaneously compensates not only for the stray capacitances and inductances, but also for the crystal’s natural temperature characteristics. Moreover, it enables additional software-managed decreasing of the inductance and any parasitic capacitance minimizing external influences.

The hysteresis measurement results clearly show that the oscillator switching method for high-precision inductance-to-frequency conversion opens up new possibilities through the compensation of the main oscillating element self-temperature and minimization of other frequency variation influences (such as aging, the influence of the supply voltage on the oscillating circuit, as well as the reference frequency temperature instability). The method can be applied in many different fields, such as nanorobotics, mechatronics, magnetic material properties measurement, biosensor technology, as well as in specific fields of physics.

## References

[1] Kenton B.J., Leang K.K. (2012). Design and control of a three-axis serial-kinematic high-bandwidth nanopositioner. IEEE/ASME Trans. Mechatron..

[2] Noth K.T., Ryu U.C., Lee Y.W. (2014). Compact and wide range polarimetric strain sensor based on polarization-maintaining photonic crystal fiber. Sens. Actuators A Phys..

[3] Chen S.C., Le D.K., Nguyen V.S. (2014). Inductive displacement sensors with a notch filter for an active magnetic bearing system. Sensors.

[4] Guo Y.X., Shao Z.B., Li T. (2016). An analog-digital mixed measurement method of inductive proximity sensor. Sensors.

[5] Xiong J., Li C., Jia P., Chen X., Zhang W., Liu J., Xue C., Tan Q. (2015). An Insertable passive lc pressure sensor based on an alumina ceramic for in situ pressure sensing in high-temperature environments. Sensors.

[6] Li C., Tan Q., Jia P., Zhang W., Liu J., Xue C., Xiong J. (2015). Review of research status and development trends of wireless passive LC resonators for harsh environments. Sensors.

[7] Chiou J.A., Chen S. (2007). Thermal hysteresis and voltage shift analysis for differential pressure sensors. Sens. Actuators A Phys..

[8] García A., Morón C., Tremp E. (2014). Magnetic sensor for building structural vibration. Sensors.

[9] Jeranče N., Bednar N., Stojanović G. (2013). An Ink-Jet Printed Eddy Current Position Sensor. Sensors.

[10] García M.H., Barrera D., Amat R., Kurlyandskaya G.V., Sales S. (2016). Magnetic actuator based on giant magnetostrictive material Terfenol-D with strain and temperature monitoring using FBG optical sensor. Measurement.

[11] Matko V., Jezernik K. (2012). New quartz oscillator switching method for nano-henry range inductance measurements. Sensors.

[12] Arnau A. (2008). A review of interface electronic systems for AT-cut quartz crystal microbalance applications in liquids. Sensors.

[13] Ferrari M., Ferrari V., Marioli D., Taroni A., Suman M., Dalcanale E. (2006). In-liquid sensing of chemical compounds by QCM sensors coupled with high-accuracy ACC oscillator. IEEE Trans. Instrum. Meas..

[14] Gagnepain J.J. (1990). Sensitivity of quartz oscillator to the environment: Characterization methods and pitfalls. IEEE Trans. Ultrason. Ferroelect. Freq. Cont..

[15] Schrüfer E. (1992). Quartz as a frequency reference. Electrical Measurement.

[16] Brice J.C. (1985). Crystals for quartz resonators. Rev. Mod. Phys..

[17] Meeker T.R. (2007). Theory and Properties of Piezoelectric Resonators and Waves. Precision Frequency Control.

[18] Yeh C.A., Lai Y.S. (2012). Digital pulsewidth modulation technique for a synchronous buck converter to reduce switching frequency. IEEE Trans. Ind. Electron..

[19] Zhao Z., Lai J.S., Cho Y. (2013). Dual-mode double-carrier-based sinusoidal pulse width modulation inverter with adaptive smooth transition control between modes. IEEE Trans. Ind. Electron..

[20] Kiatsookkanatorn P., Sangwongwanich S. (2012). A unified PWM method for matrix converters and its carrier-based realization using dipolar modulation technique. IEEE Trans. Ind. Electron..

[21] Driscoll M.M. (2008). Oscillator AM-to-FM Noise conversion due to the dynamic frequency-drive sensitivity of the crystal resonator. IEEE FCS.

[22] Filler R.L., Vig J.R. (1992). Long-term aging of the oscillators. IEEE Trans. Ultrason. Ferroelectr. Freq. Cont..

[23] Matko V., Jezernik K. (2010). Greatly improved small inductance measurement using quartz crystal parasitic capacitance compensation. Sensor.

[24] Wu I.C., Lo C.W., Fong K.L. (2008). Method and Apparatus for a Crystal Oscillator to Achieve Fast Start-Up Time, Low Power and Frequency Calibration. US Patent.

[25] Ruslan R.I., Satoh T., Akitsu T. (2012). Short-term Stability in the Intermediate Region between Quartz Crystal Oscillation and LC Oscillation. IEEJ Trans. Electr. Electron. Eng..

[26] Rutman J. (1978). Characterization of phase and frequency instabilities in precision frequency sources. Proc. IEEE.

[27] Langfelder G., Caspani A., Tocchio A. (2014). Design criteria of low-power oscillators for consumer-grade MEMS resonant sensors. IEEE Trans. Ind. Electron..

[28] Wang S., Lee F. (2010). Analysis and applications of parasitic capacitance cancellation techniques for EMI suppression. IEEE Trans. Ind. Electron..

[29] Kao P., Allara D., Tadigadapa S. (2009). Fabrication and performance characteristics of high-frequency micromachined bulk acoustic wave quartz resonator arrays. Meas. Sci. Technol..

